# Phycobiliprotein-mediated synthesis of biogenic silver nanoparticles, characterization, *in vitro* and *in vivo* assessment of anticancer activities

**DOI:** 10.1038/s41598-018-27276-6

**Published:** 2018-06-12

**Authors:** Noura El-Ahmady El-Naggar, Mervat H. Hussein, Asmaa Atallah El-Sawah

**Affiliations:** 1 Department of Bioprocess Development, Genetic Engineering and Biotechnology Research Institute, City of Scientific Research and Technological Applications, Alexandria, Egypt; 2 Botany Department, Faculty of Science, Mansoura University, Mansoura, Egypt

## Abstract

Phycoerythrin is the main phycobiliprotein that responsible for harvesting light for photosynthesis in cyanobacteria. In this research, phycoerythrin extracted from the cyanobacterium *Nostoc carneum* has been used to reduce silver nitrate for silver nanoparticles (AgNPs) biosynthesis. UV–visible spectrophotometry for measuring surface plasmon resonance showed a single absorption peak at 430 nm, which confirmed the presence of AgNPs. The face-centered central composite design was chosen to evaluate the interaction effects between four process variables and also to determine their optimal levels which influence the AgNPs biosynthesis using phycoerythrin. The maximum silver nanoparticles biosynthesis (1733.260 ± 21 µg/mL) was achieved in the central runs under the conditions of initial pH 10, incubation period of the 24 h, phycoerythrin concentration of the 0.8 mg/mL and 20 mM of AgNO_3_. The biosynthesized AgNPs were characterized using TEM which revealed the formation of spherical shape nanoparticles with size ranged between 7.1‒26.68 nm. EDX analysis confirmed silver as the major constituent element. FTIR spectrum indicates the presence of proteinaceous capping agent that prevents silver nanoparticles agglomeration. The IC_50_ of cell inhibition by AgNPs was observed at 13.07 ± 1.1 µg/mL. Treatment of mice bearing Ehrlich ascites carcinoma with 5 mg AgNPs/kg of mice body weight significantly decreased tumor volume, tumor cells count, white blood cells count and body weight. It was concluded that the phycoerythrin protein has the ability to synthesize AgNPs, which have antibacterial, antihemolytic, *in vitro* and *in vivo* cytotoxic activities.

## Introduction

Nanotechnology is the study of synthesis, design and manipulation of nanomaterials^1^. The word “nano” is derived from a Greek word meaning dwarf or extremely small, ranging in dimension from 1 to 100 nanometers. Nanoparticles have unique properties (electronic, optical and chemical properties) different from those observed with the bulk compounds. The physical characteristics of a bulk material are fixed regardless of their size, but at the nanoscale they show the most interesting characteristics, due to the fact that nanoparticles possess a very high percentage of surface area to volume ratio^2^. Recently, nanoparticles have a great effect in biomedical applications, such as biosensors, drug and gene delivery, diagnostic tools and cancer treatment which have extensively studied throughout the past decade^3^.

There are different types of nanomaterials such as magnesium, copper, gold, zinc, silver and selenium have been used nowadays, but silver is consider the most effective noble metal nanoparticle as it has good antimicrobial efficacy against fungi, viruses and bacteria^4^. There are many applications of AgNPs for example; they can be used in selective spectral coatings to absorb solar energy and intercalation material for electrical batteries, as catalysts in chemical reactions, as optical receptors, as well as for biological labeling and antimicrobials. The high antimicrobial activity of AgNPs is due to their extremely large surface area, which provides better contact and interaction with microorganisms^5^.

Nanoparticles that produced conventionally by using physical and chemical methods results in toxic byproducts that are environmental hazards. Additionally, these particles cannot be used in medicine due to health-related issues, especially in clinical fields^6^. Nanobiotechnology represents an economic alternative to chemical and physical methods for the formation of nanoparticles. In the biological synthesis of nanoparticles, the chemical reduction of the bulk material does not need extreme energy and avoids organic solvents and toxic reagents that give the toxicity properties of the nanoparticles^7^. There are different resources naturally available for the green synthesis of nanoparticles including fungi, bacteria, yeast, algae, viruses, plants^2^ or their by-products, such as proteins and lipids, with the help of various biotechnological tools. Therefore, nanobiotechnology is a promising alternate route for synthesis of biocompatible stable nanoparticles^8^.

Many scientists use cyanobacteria in production of nanoparticles (Pt, Pd, Ag and Au) either intracellulary or extracellulary^9^. Mubarak *et al*.^10^ has used *Oscillatoria willei* NTDM01 for green synthesis of AgNPs. Cyanobacterial extracts like polysaccharides and phycocyanin used for production of AgNPs by Patel *et al*.^11^.

The purpose of this work was to apply phycobiliprotein for silver nanoparticles synthesis, to optimize the synthesis of silver nanoparticles by using phycoerythrin extracted from cyanobacterium *Nostoc carneum* using FCCD (face-centered central composite design), to characterize the synthesized AgNPs. *In vitro* cytotoxic effect against normal and human breast cancer (MCF-7) cell lines and a more interesting, *in vivo* cytotoxic effect against Ehrlich Ascites Carcinoma (EAC) have been evaluated. Further, the stability and the degree of dispersion of synthesized AgNPs have been analyzed.

## Results and Discussion

### Spectroscopic characterization of phycoerythrin

**Figure 1:**
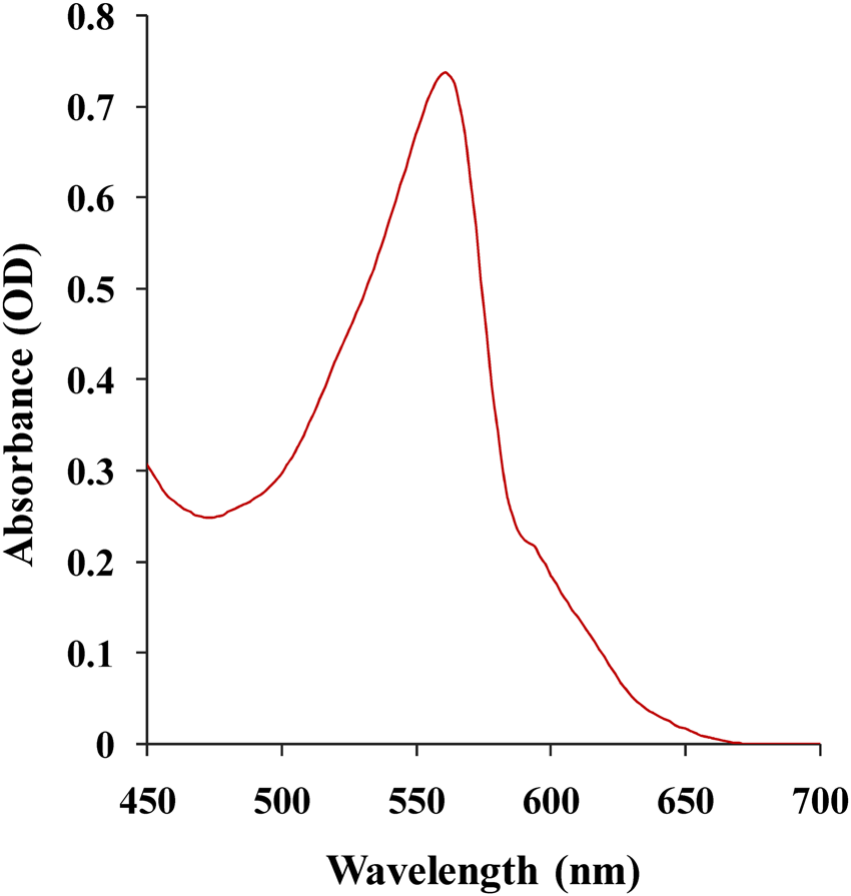
UV–Vis absorption spectrum of phycoerythrin pigment.

Phycobiliproteins are chromophored water soluble photosynthetic proteinacous pigments that are found in cyanobacteria and take over wide solar spectrum from 450 to 650 nm^12^. Phycobiliproteins are classified based on their spectral properties. Phycoerythrin (PE, λ_A max_ = 540–570 nm), phycocyanin (PC, λ_A max_ = 610–620 nm) and allophycocyanin (APC, λ_A max_ = 650–655 nm) which are the mostly detected phycobiliproteins^13^. In the present study, phycoerythrin extracted from *Nostoc carneum*  has λ_A max_ = 560 nm that displayed in Fig. 1

### AgNPs synthesis by phycoerythrin

**Figure 2:**
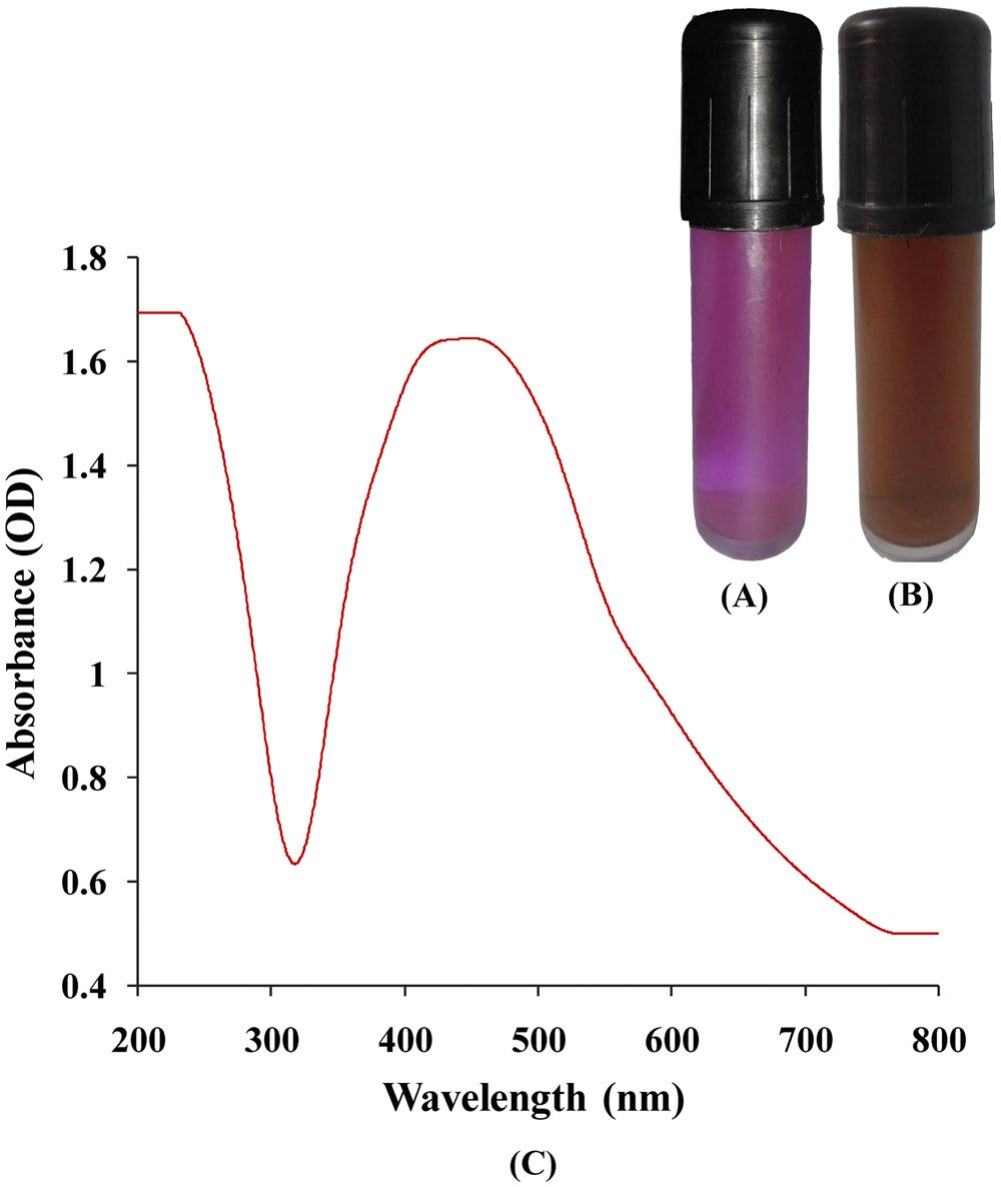
(**A**) phycoerythrin pigment; (**B**) visible observation of AgNPs biosynthesis by phycoerythrin pigment after exposure to AgNO_3_ solution (20 mM); (C) UV–Vis absorption spectrum of silver nanoparticles synthesized by phycoerythrin pigment.

Silver nanoparticles biosynthesis was performed by addition of 1 mL of 0.8 mg/mL of lyophilized phycoerythrin solution to 19 mL (20 mM) of aqueous AgNO_3_ and kept in illumination conditition. The pink color of phycoerythrin reaction mixture turned into dark brown color which demonstrating AgNPs formation (Fig. 2A,B). Formation of a dark brown color depends on the provocation of the surface plasmon vibrations of AgNPs^14^. In contrast, there was no color change detected in AgNO_3_ solution incubated in absence of phycoerythrin within similar circumstance.

### UV absorbance of AgNPs

The formation of AgNPs was confirmed by using a UV-visible spectral scan at a range of 200–800 nm. The surface plasmon resonance (SPR) spectrum of AgNPs produced by using phycoerythrin showed a distinct absorption peak at 430 nm (Fig. 2C) which confirm the presence of silver nanoparticles. Brause *et al*.^15^ reported that optical absorption spectra of metallic nanoparticles are predominantly dominated by surface plasmon resonance and the peak of absorption correlated with particle size. The SPR peak of AgNPs in aqueous dispersion shifts to longer wavelengths with increase in particle size. The position and shape of plasmon absorption of AgNPs depends on particle size, shape and the dielectric constant of the reaction medium as well as particles adsorbed on AgNPs surfaces^16^.

### AgNPs biosynthesis optimization using face-centered central composite design (FCCD)

The FCCD was employed to study the interactions among the tested variables and to determine their optimal levels. In the present study, 30 experimental runs with various combinations of X_1_ which represented the pH initial level, X_2_ which represented the concentration of AgNO_3_, X_3_ which represented phycoerythrin pigment concentration and X_4_ which represented the incubation period were carried out and the results of the experimental runs are presented along with predicted responses and residuals in Table 1. The different coded and actual levels of the four independent variables also presented in Table 1. The six times repeated central points were represented in runs (4, 5, 14, 23, 26, and 27). The results show considerable variation in the biosynthesis of AgNPs. The AgNPs maximum biosynthesis (1733.260 ± 21 µg/mL) was achieved in the runs numbers 4, 5, 14, 23, 26, and 27 which was attained at an initial pH 10, phycoerythrin concentration was 0.8 mg/mL, the concentration of AgNO_3_ was 20 mM, and the incubation period was 24 h, while the minimum silver nanoparticles biosynthesis (25.617 ± 19 µg/mL) was achieved in the run number 6 at an initial pH 5, the concentration of AgNO_3_ was 50 mM, phycoerythrin concentration was 1.6 mg/mL and the incubation period was 72 h.

**Table 1:** Face-centered central composite design matrix of four process variables with actual factor levels corresponding to coded factor levels, mean experimental and predicted values of silver nanoparticles biosynthesis by phycoerythrin pigment.

Std	Run	Type	Initial pH level (X_1_)		AgNO_3_ concentration (X_2_)		Phycoerythrin pigment concentration (X_3_)		Incubation period (X_4_)		AgNPs (µg/mL)		Residuals
			Coded	Actual	Coded	Actual (mM)	Coded	Actual (mg/mL)	Coded	Actual (h)	Experimental	Predicted	
10	1	Factorial	1	12	−1	1	−1	0.4	1	72	297.312	302.611	−5.300
6	2	Factorial	1	12	−1	1	1	1.6	−1	2	157.090	169.900	−12.810
16	3	Factorial	1	12	1	50	1	1.6	1	72	200.316	340.014	−139.698
25	4	Center	0	10	0	20	0	0.8	0	24	1733.260	1660.874	72.389
28	5	Center	0	10	0	20	0	0.8	0	24	1733.260	1660.874	72.389
15	6	Factorial	−1	5	1	50	1	1.6	1	72	25.617	−42.411	68.028
13	7	Factorial	−1	5	−1	1	1	1.6	1	72	86.452	67.986	18.466
20	8	Axial	0	10	1	50	0	0.8	0	24	696.890	802.427	−105.538
24	9	Axial	0	10	0	20	0	0.8	1	72	1721.670	1672.714	48.952
22	10	Axial	0	10	0	20	1	1.6	0	24	1698.110	1764.059	−65.948
8	11	Factorial	1	12	1	50	1	1.6	−1	2	696.828	565.904	130.924
2	12	Factorial	1	12	−1	1	−1	0.4	−1	2	43.226	60.054	−16.828
4	13	Factorial	1	12	1	50	−1	0.4	−1	2	449.130	482.601	−33.471
26	14	Center	0	10	0	20	0	0.8	0	24	1733.260	1660.874	72.389
7	15	Factorial	−1	5	1	50	1	1.6	−1	2	216.131	225.836	−9.705
11	16	Factorial	−1	5	1	50	−1	0.4	1	72	74.855	77.050	−2.195
12	17	Factorial	1	12	1	50	−1	0.4	1	72	600.387	534.423	65.964
14	18	Factorial	1	12	−1	1	1	1.6	1	72	160.253	134.745	25.508
21	19	Axial	0	10	0	20	−1	0.4	0	24	1690.040	1768.867	−78.830
18	20	Axial	1	12	0	20	0	0.8	0	24	1122.130	1136.415	−14.289
1	21	Factorial	−1	5	−1	1	−1	0.4	−1	2	85.398	−39.295	124.693
3	22	Factorial	−1	5	1	50	−1	0.4	−1	2	93.277	67.585	25.691
30	23	Center	0	10	0	20	0	0.8	0	24	1733.260	1660.874	72.389
5	24	Factorial	−1	5	−1	1	1	1.6	−1	2	130.733	145.497	−14.765
17	25	Axial	−1	5	0	20	0	0.8	0	24	765.039	895.528	−130.489
29	26	Center	0	10	0	20	0	0.8	0	24	1733.260	1660.874	72.389
27	27	Center	0	10	0	20	0	0.8	0	24	1733.260	1660.874	72.389
23	28	Axial	0	10	0	20	0	0.8	−1	2	1491.830	1685.559	−193.730
9	29	Factorial	−1	5	−1	1	−1	0.4	1	72	81.181	160.905	−79.724
19	30	Axial	0	10	−1	1	0	0.8	0	24	607.112	646.353	−39.240

### Multiple regression analysis & ANOVA

Design Expert software (Version 7) for Windows was used to perform statistical analysis for data which are represented in Tables 2 and 3. The determination coefficient (R^2^) of the model was 0.9873 (Table 2) evidence that 98.73% of variation in the biosynthesis of AgNPs was attributed to the independent variables and 1.27% only of the variation could not be explained by the model. “A regression model having an R^2^-value higher than 0.9 was considered as having a very high correlation^17^. The highest R^2^ value showed the good agreement between the experimental results and the theoretical values predicted by the model^18^.” The perfect compatibility between the predicted and observed values of silver nanoparticles indicated by reasonable agreement between the “Pred R-Squared” of 0.9340 and the “Adj R-Squared” of 0.9755. “Adeq Precision” ratio of 23.1945 indicates an adequate signal to noise ratio. Value of PRESS is 952250.03 and C.V. with lower value (14.04) indicated a better precision of the experimental performance^19^. The model used shows mean value and standard deviation of 786.35 and110.44; respectively (Table 2).

**Table 2:** Regression statistics and analysis of variance (ANOVA) for face-centered central composite design experimental values of silver nanoparticles biosynthesis by phycoerythrin pigment.

Source	Sum of Squares	*df*	Mean Square	*F-*value	*P-*value *P*rob > *F*	Confidence Level
Model	14248557.09	14	1017754.08	83.45	<0.0001*	99.99
X_1_	261120.25	1	261120.25	21.41	0.0003*	99.97
X_2_	109617.16	1	109617.16	8.99	0.0090*	99.10
X_3_	104.03	1	104.03	0.01	0.9276	7.24
X_4_	742.45	1	742.45	0.06	0.8085	19.15
X_1_ X_2_	99645.18	1	99645.18	8.17	0.0120*	98.80
X_1_ X_3_	5617.10	1	5617.10	0.46	0.5077	49.23
X_1_ X_4_	1794.09	1	1794.09	0.15	0.7067	29.33
X_2_ X_3_	704.49	1	704.49	0.06	0.8133	18.67
X_2_ X_4_	36379.97	1	36379.97	2.98	0.1047	89.53
X_3_ X_4_	77123.72	1	77123.72	6.32	0.0238*	97.62
X_1_^2^	1077557.63	1	1077557.63	88.35	<0.0001*	99.99
X_2_^2^	2272233.89	1	2272233.89	186.30	<0.0001*	99.99
X_3_^2^	28885.83	1	28885.83	2.37	0.1446	85.54
X_4_^2^	864.09	1	864.09	0.07	0.7937	20.63
Residual	182945.59	15	12196.37			
Lack of Fit	182945.59	10	18294.56			
Pure Error	0	5	0			
Cor Total	14431502.67	29				
Std. Dev.	110.44	R-Squared		0.9873		
Mean	786.35	Adj R-Squared		0.9755		
C.V.%	14.04	Pred R-Squared		0.9340		
PRESS	952250.03	Adeq Precision		23.1945		

*Significant values, *df*: Degree of freedom, *F*: Fishers’s function, *P*: Level of significance, C.V: Coefficient of variation.

**Table 3:** Regression coefficients of second order polynomial model for optimization of silver nanoparticles biosynthesis by phycoerythrin pigment.

Factor	Coefficient estimate	*df*	Standard error	95% CI Low	95% CI High
Intercept	1660.87	1	34.31	1587.75	1733.99
X_1_	120.44	1	26.03	64.96	175.93
X_2_	78.04	1	26.03	22.56	133.52
X_3_	−2.40	1	26.03	−57.89	53.08
X_4_	−6.42	1	26.03	−61.90	49.06
X_1_ X_2_	78.92	1	27.61	20.07	137.76
X_1_ X_3_	−18.74	1	27.61	−77.58	40.11
X_1_ X_4_	10.59	1	27.61	−48.26	69.44
X_2_ X_3_	−6.64	1	27.61	−65.48	52.21
X_2_ X_4_	−47.68	1	27.61	−106.53	11.16
X_3_ X_4_	−69.43	1	27.61	−128.28	−10.58
X_1_^2^	−644.90	1	68.61	−791.14	−498.66
X_2_^2^	−936.48	1	68.61	−1082.72	−790.24
X_3_^2^	105.59	1	68.61	−40.65	251.83
X_4_^2^	18.26	1	68.61	−127.98	164.50

*Significant values, *df*: Degree of freedom.

**Table 4:** Antibacterial activity of silver nanoparticles produced by using phycoerythrin pigment against bacterial species.

Microorganism	Zone of inhibition (mm)		
	Vancomycin	AgNPs	AgNPs + Vancomycin
*E. coli*	20	21	25
*Staphylococcus aureus*	20	16	23
*Enterobacter aerogenes*	20	18	25
*Streptococcus* sp.	22	18	27

The Model *F*-value of 83.45 with a very low probability value (*P*-value (Prob > *F*) <0.0001) implies the model is significant. The significance degrees (Table 2) indicated that the linear coefficients of initial pH level (X_1_), AgNO_3_ concentration (X_2_), interaction between initial pH level (X_1_), AgNO_3_ concentration (X_2_), interaction between phycoerythrin concentration (X_3_) and incubation period (X_4_) and also quadratic effect of initial pH level (X_1_), AgNO_3_ concentration (X_2_) are significant model terms. The *P-* values of the coefficients indicated that among the four factors, initial pH level (X_1_), AgNO_3_ concentration (X_2_) shows maximum interaction (*P-*value 0.0120) between the two factors and 98.80% of the model was influenced by the interaction between these two factors. However, the linear coefficients of phycoerythrin concentration (X_3_) and incubation period (X_4_) are not significant. Furthermore, among the different interactions, interaction between X_1_ and X_3_, X_1_ and X_4_, X_2_ and X_3,_ X_2_ and X_4_ are insignificant (*P-* value > 0.05).

The positive coefficients for X_1_ and X_2_ (linear coefficient), X_1_ X_2_ and X_1_ X_4_ (interaction coefficients), in addition of X_3_^2^ and X_4_^2^ (quadratic coefficients) indicates the increase in AgNPs biosynthesis (Table 3). In contrast, the negative coefficients indicate the decline in biosynthesis of AgNPs.

A second-order polynomial model was proposed to evaluate the relationship between both dependent and independent factors and to determine the maximum AgNPs biosynthesis corresponding to the optimum levels of initial pH level, AgNO_3_ concentration, phycoerythrin concentration and incubation period. Using the multiple regression analysis on experimental data, the second-order polynomial equation that determines the expected response (Y) was obtained in terms of the independent factors:

1 $${\rm{Y}}=1660.87+120.44\,{{\rm{X}}}_{1}+78.04\,{{\rm{X}}}_{2}-2.40\,{{\rm{X}}}_{3}-6.42\,{{\rm{X}}}_{4}+78.92\,{{\rm{X}}}_{1}\,{{\rm{X}}}_{2}-18.74\,{{\rm{X}}}_{1}\,{{\rm{X}}}_{3}\,+10.59\,{{\rm{X}}}_{1}\,{{\rm{X}}}_{4}-6.64\,{{\rm{X}}}_{2}\,{{\rm{X}}}_{3}-47.68\,{{\rm{X}}}_{2}\,{{\rm{X}}}_{4}-69.43\,{{\rm{X}}}_{3}\,{{\rm{X}}}_{4}-644.90\,{{\rm{X}}}_{1}^{2}-936.48\,{{\rm{X}}}_{2}^{2}\,+105.59\,{{\rm{X}}}_{3}^{2}+18.26\,{{\rm{X}}}_{4}^{2}$$

Where Y is AgNPs biosynthesis (the response) and X_1_, X_2_, X_3_ and X_4_ are initial pH level, concentration of AgNO_3_, concentration of phycoerythrin pigment and incubation period; respectively.

### Investigation of polynomial model accuracy

**Figure 3:**
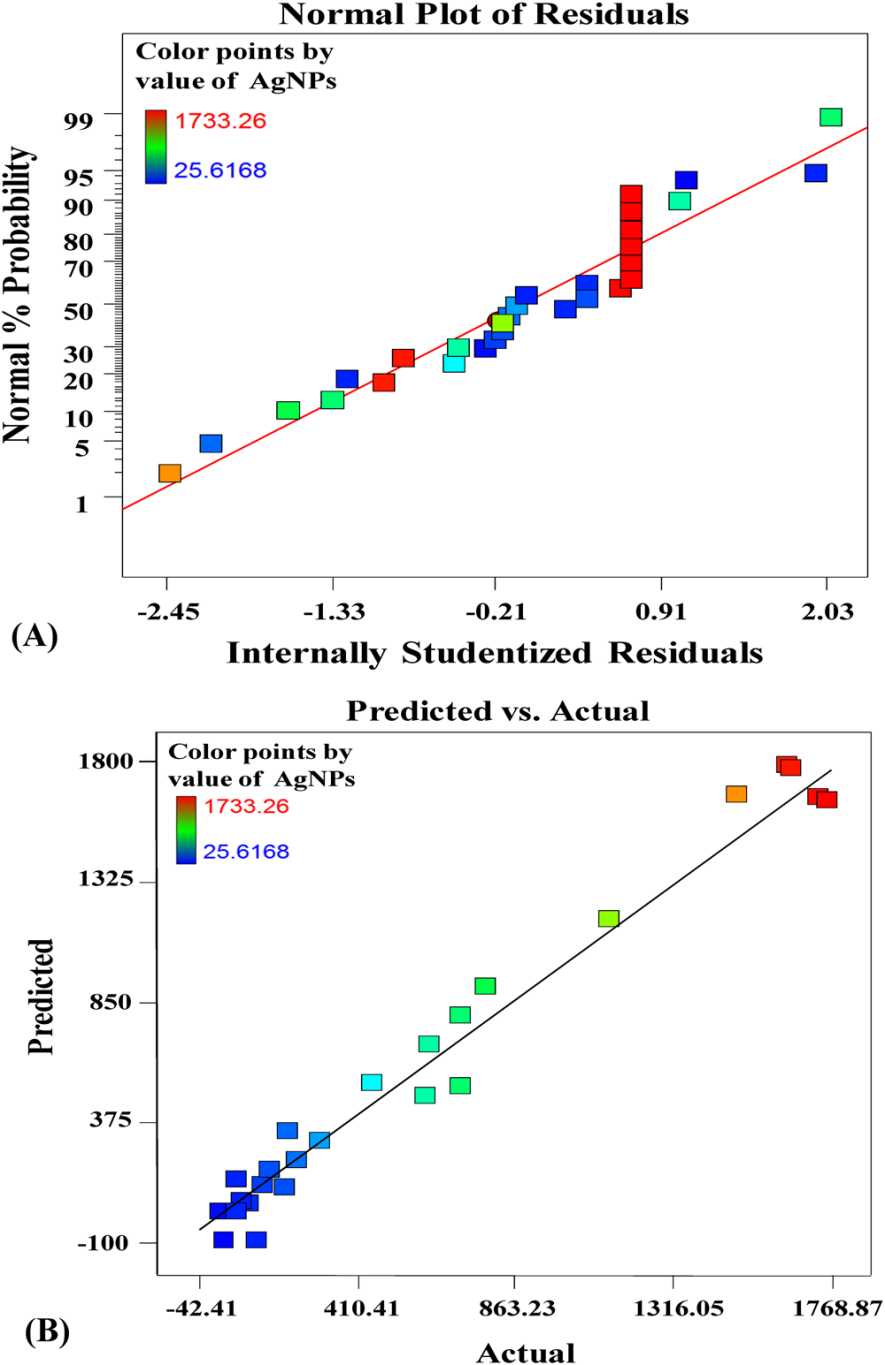
(**A**) The normal probability plot of the residuals. (**B**) Correlation between the experimented and predicted values for silver nanoparticles biosynthesis using phycoerythrin pigment determined by the second-order polynomial equation.

The normal probability plot is an important tool for assessing whether the residuals are normally distributed. Figure 3A showed that the residuals were normally distributed a long a straight line for AgNPs biosynthesis, this indicates that the model had been validated. Also, predicted versus actual AgNPs biosynthesis plot confirmed that, there is a close agreement between the theoretical expected values by the model equation and the experimental values as shown in Fig. 3B which confirms the accuracy of the second-polynomial model.

### Three dimensional (3D) plots

Not only the variables interaction effects were determined, but also their optimal levels by plotting the response surface curves for the pair-wise combinations between the variables (X_1_ X_2_, X_1_ X_3_, X_1_ X_4_, X_2_ X_3_, X_2_ X_4_ and X_3_ X_4_**)**, the AgNPs biosynthesis was plotted on Z-axis versus two independent factors and keeping the other two factors at their center (zero) levels to detect the optimal conditions for AgNPs biosynthesis by using phycoerythrin.

**Figure 4:**
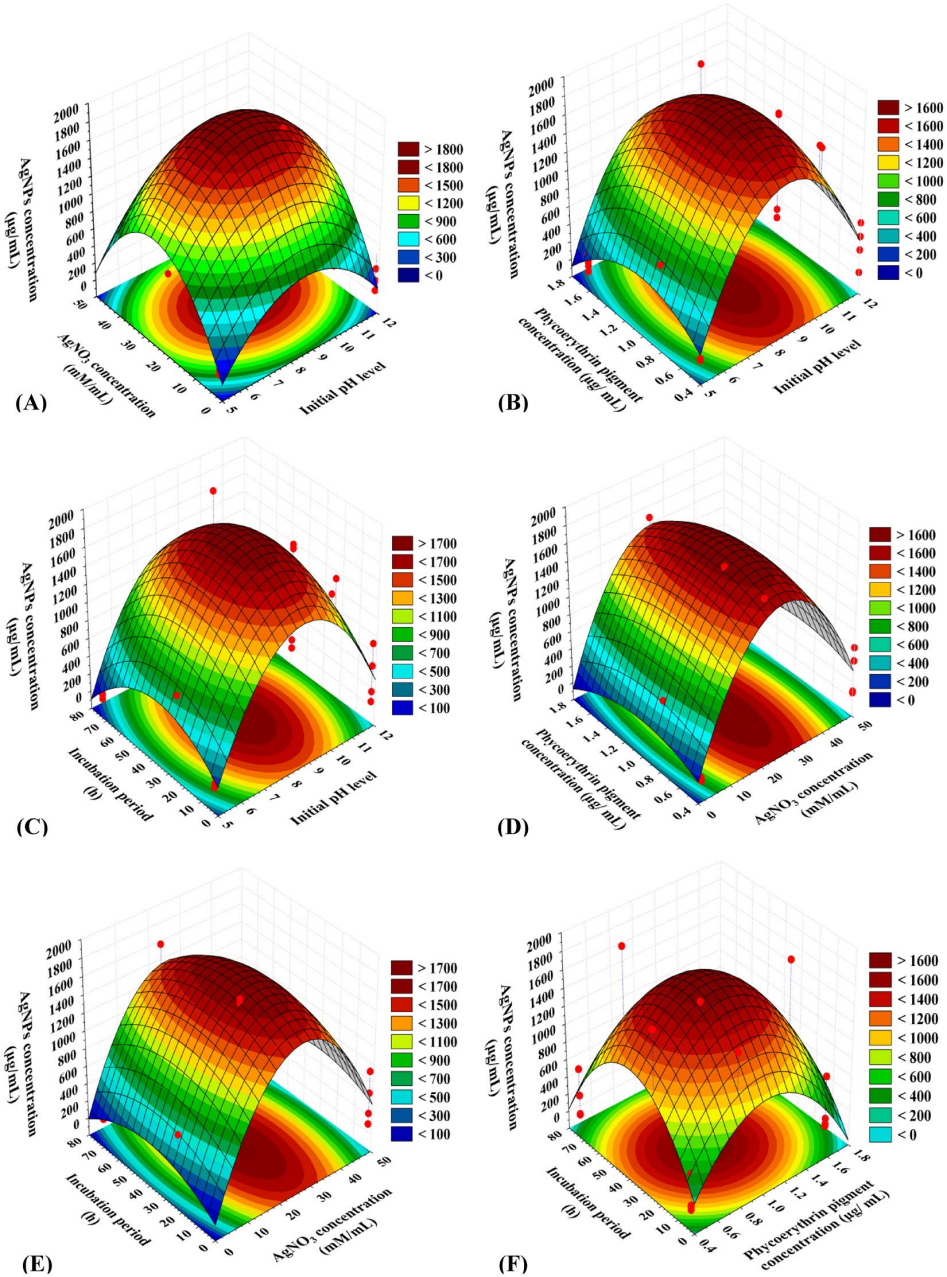
Three-dimensional response surface plots (**A**–**F**) showing the interactive effects of independent variables: initial pH level, AgNO_3_ concentration, phycoerythrin pigment concentration and incubation period on biosynthesis of silver nanoparticles.

Figure 4A represents the interaction between AgNO_3_ concentration and initial pH level, while the other two variables were remained at their central or zero values; phycoerythrin concentration (X_3_) was 0.8 mg/mL and incubation period (X_4_) was 24 h. It was observed that the maximum AgNPs yield obtained within alkaline initial pH.

The size of nanoparticles and the distribution are affected by the physical factor pH^20^. Basic pH induced perfect yield, rapid growth rate and mono dispersity of AgNPs as reported by Khalil *et al*.^21^. The milk protein casein was used to synthesize greatly stable silver nanoparticles with no supplementation of other reducing agents as documented by Ashraf *et al*.^22^ They found that these biosynthesized AgNPs go through reversible sedimentation developing protein–silver nanoparticle composite precipitates when pH was around the isoelectric point of casein protein (pH = 4.6). They also reported that the produced silver nanoparticles sizes using the protein of casein milk ranged between 3 to 18 nm when pH was >7 and when pH was <6, the silver nanoparticles sizes ranged between 60 to 80 nm. Ortega-Arroyo *et al*.^23^ investigated usage of polysaccharide starch as capping agent in the green synthesis of the nanoparticles. They found that small spherical nanoparticles size were fabricated at pH = 11. On the other hand, El-Rafie *et al*.^24^ elucidated that spherical sized AgNPs of 7–20 nm were formed in crude hot water soluble polysaccharide solutions extracted from some marine macroalgae (*Pterocladia capillacae*, *Jania rubins*, *Ulva faciata* and *Colpmenia sinusa*) as bio-reducing and stabilizing factors for AgNPs biosynthesis when reacted with 0.1 mM AgNO_3_ in pH 10 and also confirmed that the basic medium is the best medium to biosynthesize AgNPs which agreed with our results (pH = 10).

It was observed that the AgNPs synthesis using phycoerythrin extracted from cyanobacterium *Nostoc carneum* was suppressed by acidic conditions at pH 5 and enhanced by alkaline conditions at pH 10, suggesting that the reduction rate of silver ions increases with an increase in pH. At pH 10, smaller (size ranged between 7.1 to 26.68 nm), and highly dispersed AgNPs were formed. Whereas, at higher pH (pH 12), larger nanoparticles were formed, aggregated, precipitated and instable with size ranged between 18.72 to 54.84 nm. Tagad *et al*.^25^ reported that the aggregation and instability of the biologically synthesized AgNPs using locust bean gum (LBG) polysaccharide appear at high pH (pH > 11). Most of the researchers prefer neutral medium for the biosynthesis of AgNPs. Using ethyl acetate extract of *Ulva fasciata* as reducing agent for the biosynthesis of AgNPs at neutral pH, spherical shape AgNPs with size ranged between 28 to 41 nm were produced as indicated by Rajesh *et al*.^26^.

On the other hand, acidic pH has been used in silver nanoparticles biosynthesis but in a little frequency. Bankar *et al*.^27^ used acidic medium in AgNPs biosynthesis using angiosperms, AgNPs have face-centered cubic structure with size <100 nm in pH medium ranged between 2–5 using acetone extract of banana peel powder. Ultimately, basic pH is the best for biosynthesis of silver nanoparticles as reported by El-Rafie *et al*.^24^ which is in agreement with our results.

As shown in Fig. 4A, AgNPs biosynthesis increases gradually with the increase in the concentration of AgNO_3_ till reach a certain point then any further increase in AgNO_3_ concentration resulted in gradual decline in AgNPs biosynthesis. By solving the Equation (1) and analysis of Fig. 4A, the maximum AgNPs synthesis of 1740 µg/mL was obtained at the optimum predicted levels of initial pH 10 and 27 mM of AgNO_3_ at phycoerythrin concentration of 0.8 mg/mL and incubation period of 24 h. The Ag^+^ ions reduction to Ag° nanoparticles was designated by altering the solution mixture color from yellow to dark-brown depending on the reducing factor and AgNO_3_ concentrations. The researchers use a broad range of AgNO_3_ concentrations from 0.1 mM to 1 M to synthesize AgNPs. 1 mM of AgNO_3_ is the most widespread concentration used for the AgNPs biosynthesis as used with aqueous extract of *Plectranthus amboinicus* leaf by Ajitha *et al*.^28^. Kathiraven *et al*.^29^ also used 10^−3^ M (1 mM) of AgNO_3_ solution with *Caulerpa resmosa* extract for synthesis of silver nanoparticles. 1 mM AgNO_3_ has been also used in silver nanoparticles biosynthesis using a nanofactory *Streptomyces narbonensis* SSHH-1E by El-Naggar *et al*.^30^. Sadeghi and Gholamhoseinpoor^31^ have used 0.1 mM of AgNO_3_ in AgNPs biosynthesis by *Ziziphora tenuior* leaf methanol extract and also 500 mM of AgNO_3_ was used in AgNPs biosynthesis by using methanol extract of *Viburnum lantana* leaf evaporated in vacuo^32^. Veerasamy *et al*.^33^ used filtrate of *Garcinia mangostana* leaf as reducing agent by adding of 5 mM of AgNO_3_. Also high concentration of AgNO_3_ (1 M) has been used by Gnanadesigan *et al*.^34^ in the biosynthesis of AgNPs using dextran.

Figure 4B demonstrates the interaction between phycoerythrin pigment concentration (X_3_) and initial pH level (X_1_), while the other two variables were remained at their central or zero values; AgNO_3_ concentration (X_2_) was 20 mM and incubation period (X_4_) was 24 h. It was observed that highest yield of AgNPs were achieved within alkaline initial pH. The biosynthesis of AgNPs gradually increases with the increase in phycoerythrin concentration till reach a certain point then any rising in the concentration of phycoerythrin resulted in the gradually decline in the biosynthesis of AgNPs. By solving the Equation (1) and analysis of Fig. 4B, the maximum AgNPs synthesis of 1839 µg/mL was obtained at the optimum predicted levels of initial pH 9 and phycoerythrin concentration of 0.4 mg/mL at 20 mM of AgNO_3_ and incubation period of 24 h. Patel *et al*.^11^ used cyanobacteria in biosynthesis of AgNPs. They extracted the phycobiliproteins and polysaccharides and used them as reducing agents to synthesize silver nanoparticles.

Figure 4C demonstrates the interaction between pH initial level (X_1_) and incubation period (X_4_), while the other two variables were remained at their central or zero values; AgNO_3_ concentration (X_2_) was 20 mM and phycoerythrin concentration (X_3_) was 0.8 mg/mL. It was observed that AgNPs highest yield were achieved within alkaline initial pH. Furthermore, the highest AgNPs yield was found at the central value of incubation period (X_4_).

The formation of AgNPs using *Anabaena doliolum* was observed visually after 2 h by the changing in the original color of the mixture that was reddish blue due to the fluorescence of phycobiliprotein to brown according to Singh *et al*.^35^. Mohseniazar *et al*.^36^ studied the biosynthesis of AgNPs using microalgae *Nannochloropsis oculata* and *Chlorella vulgaris* and they documented that the two algal species have the potential of AgNPs biosynthesis which occurred in the culture medium using 1 mM of AgNO_3_ and the proper incubation time was 24 h. Incubation of the microalgae for 48 h had no significant effect on the production of silver nanoparticles. The tendency of AgNPs to agglomerate after certain reaction time increases because of the formation of larger nanoparticles^5^.

Figure 4D represents the interaction between the concentration of AgNO_3_ (X_2_) and the concentration of phycoerythrin (X_3_), while the other two variables were remained at their central or zero values; pH initial level (X_1_) was 10 and incubation period (X_4_) was 24 h. It was observsed that the highest AgNPs yield was found at the central values of both AgNO_3_ concentration (X_2_) and phycoerythrin concentration (X_3_).

Figure 4E exhibits the interaction between incubation period (X_4_) and the concentration of AgNO_3_ (X_2_) on the biosynthesis of AgNPs, while the other two variables were remained at their central or zero values; pH initial level (X_1_) was 10 and phycoerythrin concentration (X_3_) was 0.8 mg/mL. It was observed that the highest AgNPs yield was found at the central values of both incubation period (X_4_) and the concentration of AgNO_3_ (X_2_).

Figure 4F highlight the roles played by phycoerythrin concentration (X_3_) and incubation period (X_4_) on AgNPs boisynthesis while the other two variables were remained at their central or zero values; pH initial level (X_1_) was 10 and the concentration of AgNO_3_ (X_2_) was 20 mM. In addition, Fig. 4F clearly demonstrates that as phycoerythrin concentration and incubation period increased, AgNPs biosynthesis increase, but further increase in both phycoerythrin concentration and incubation period resulted in decreasing in the biosynthesis of AgNPs.

### The polynomial model verification

Experiments with optimum conditions were repeated three times and compared to their predicted values by second-order polynomial equation for detecting the adequacy of the model and to verify the optimum concentrations of the variables. Predicted value of silver nanoparticles biosynthesis was 1660.874 µg/mL while the experimental value was 1733.260 ± 21 µg/mL. Therefore, it was observed that the model verification exhibited high degree of accuracy under the optimized condition equaled 95.82%.

### Characterization of the biosynthesized AgNPs using phycoerythrin

#### Transmission electron microscopy (TEM)

**Figure 5:**
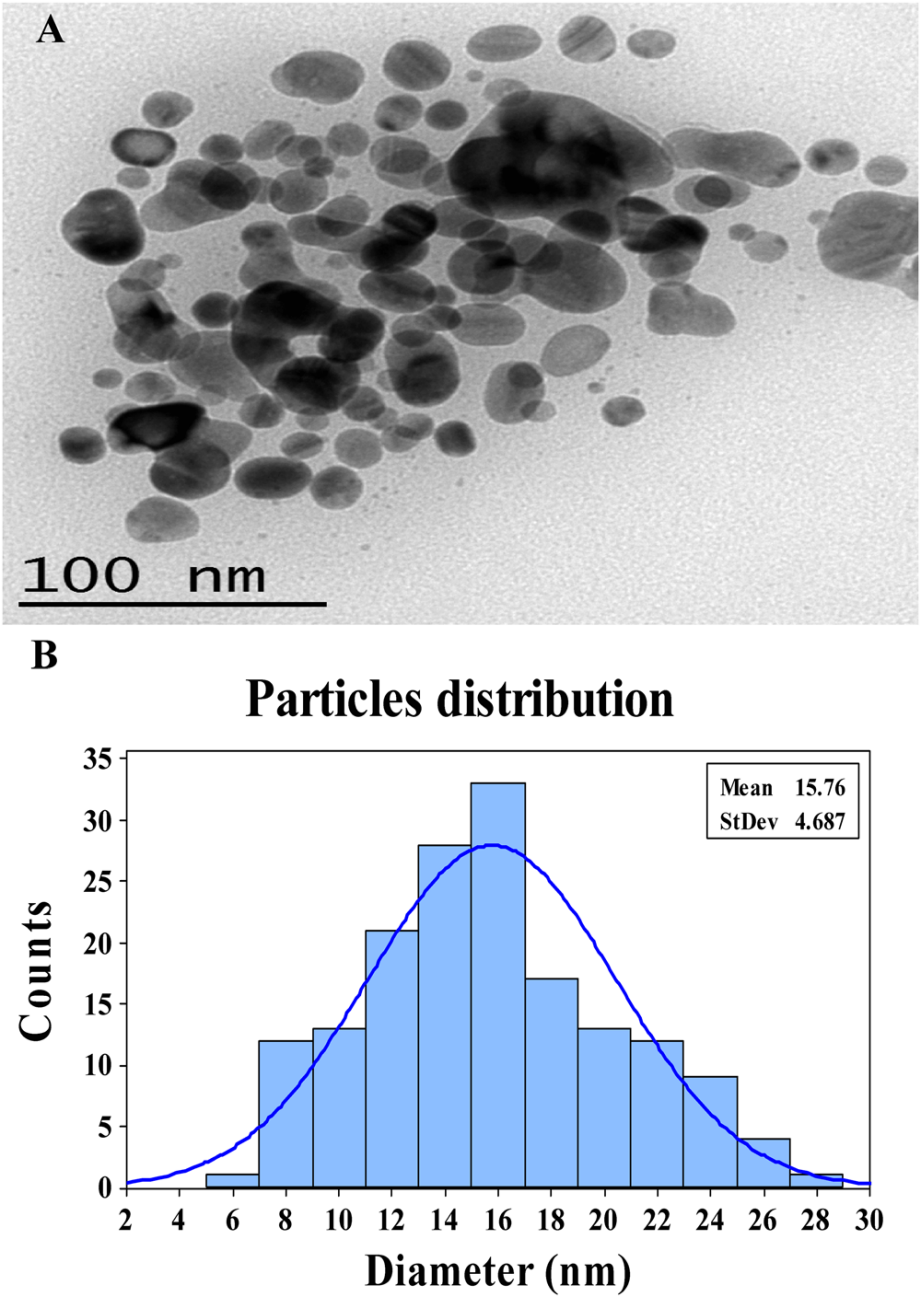
(**A**) Transmition electron microscopy image of produced silver nanoparticles using phycoerythrin pigment; (**B**) Particle size distributions after 1 day of synthesis.

TEM is a powerful method for detecting the morphology and size of nanostructures. TEM revealed that the synthesized AgNPs have spherical shapes with dimensions ranged from 7.1 to 26.68 nm (Fig. 5). One hundred sixty four nanoparticles were used to calculate the mean size of particles (after 1 day of synthesis) which was found to be 15.76. The microbial mediated biosynthesized AgNPs mostly spherical in shape. Kathiraven *et al*.^29^ documented that AgNPs have “spherical shape face-centered cubic structure” with size ranged between 5–25 nm.

#### Energy-dispersive X-ray (EDX)

**Figure 6:**
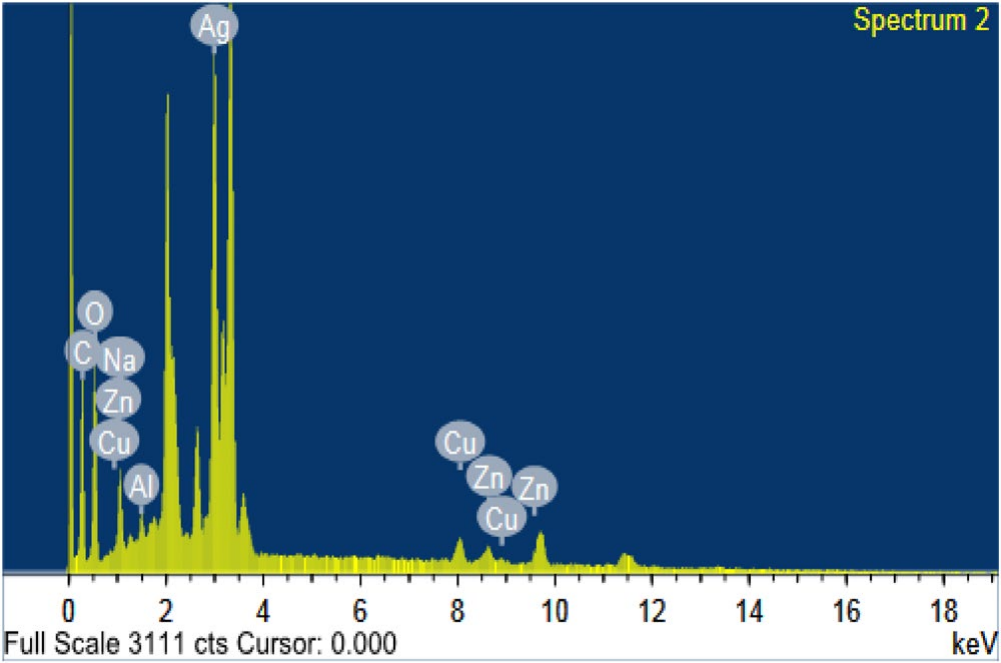
EDX spectrum showing peak between 3 and 4 keV confirming the presence of silver.

EDX analysis is used to detect all the constituent elements and the EDX spectrum showing peak between 3 and 4 keV confirming the presence of silver (Fig. 6). The spectrum graph at 3 keV indicates that the silver has an intensive signal which is characteristic to AgNPs^37^ with weight percentage 20.49% and atomic percentage of 3.46%. EDX spectra recorded from the silver nanoparticles revealed the silver nanoparticles reduced by *Catharanthus roseus* have the weight percentage of silver as 20.16% and atomic percentage of 16.41%^38^. In addition, EDX analysis confirmed high atomic and weight percentage of carbon and oxygen which are possibly due to emissions from proteins^39^. Peaks for Na, Cu, Zn and Al were also observed. Cyanobacteria are a rich source of numerous microelements such as Ca, Fe, P, I, Mg, Zn, Se, Cu, Mn, Cr, K, and Na^40^. Thus, Na, Cu and Zn were leaked out from the cyanobacterium during the extraction of the phycoerythrin. Also, the presence of aluminum is due to aluminum grid sample holder used to support the sample.

#### Fourier transformed infrared (FTIR) spectroscopy analysis

**Figure 7:**
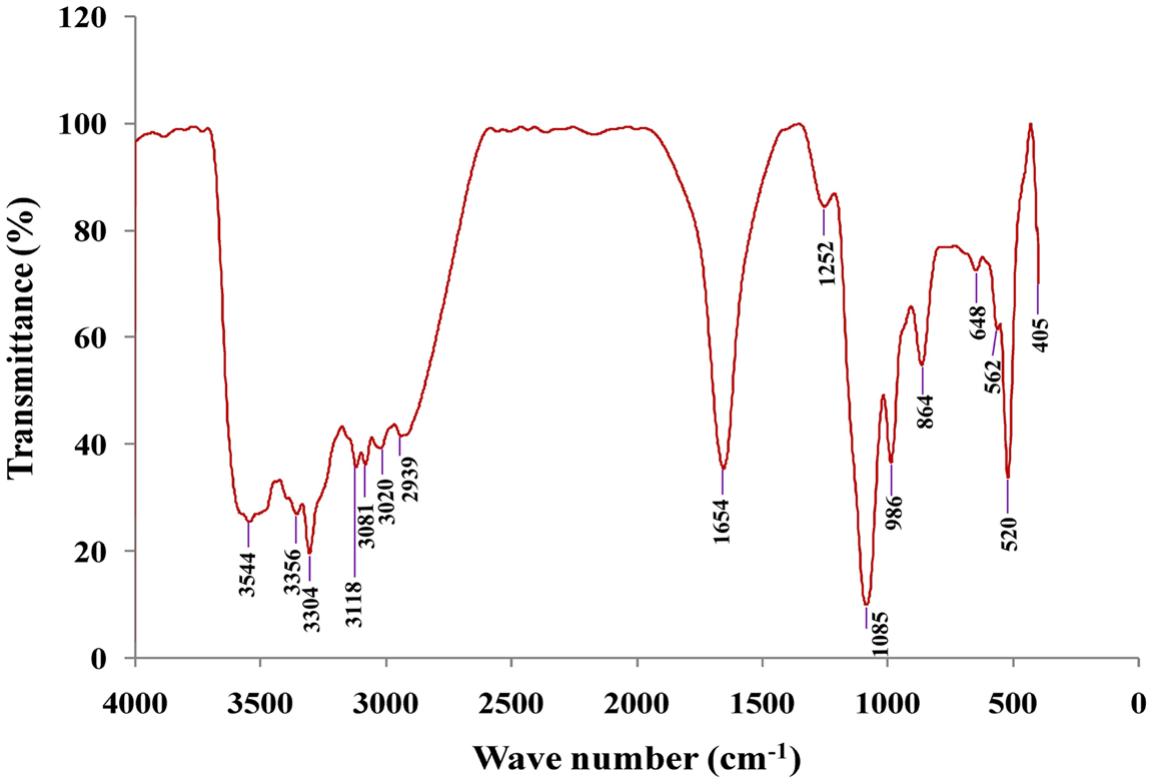
FTIR spectrum recorded by making KBr disc with synthesized silver nanoparticles by using phycoerythrin pigment.

FTIR  analysis was carried out to identify the possible potential biomolecules responsible for the reduction of the Ag^+^ ions and capping of the bio-reduced silver nanoparticles^41^. FTIR spectrum of the obtained AgNPs (Fig. 7) manifests 15 absorption peaks at 3544, 3356, 3304, 3118, 3081, 3020, 2939, 1654, 1252, 1085, 986, 864, 648, 562, 520 and 405 cm^−1^. The absorption peak at 3544 cm^−1^ is referring to the stretching vibration of hydroxyl group. The peak at 3356 cm^−1^ expresses the stretching bond of amide linkages in proteins^42^. In AgNPs FTIR, the bands at 3304 cm^−1^ in addition to 1637 cm^−1^ are attributed to the hydroxyl group and binding of C = O functional group^26^.

The emergence of signal in FTIR spectrum at 3118 cm^−1^ expresses aromatic C-H groups. The peak at 3061 cm^−1^ expresses a stretching vibration of –C–H bonds existed in rings. The peak at 3020 cm^−1^ expresses O–H group of either alcohols or phenols revealing that phenols has played a role in reducing the contents of nanoparticles^43^. The presence of weak peak at 2939 cm^−1^ expresses C-H stretching alkanes^44^. The peak at 1654 cm^−1^ is referring to the existence of C–O stretching in carboxyl linked to the amide linkage in amide I^45^. Lambert *et al*.^46^ reported that the peak at 1654 cm^−1^ is referring to N–H deformation, C = N and C = O which present in “N–H primary amides II, Oxime and C = O in carbonyl compound”; respectively. The existence of 1252 cm^−1^ peak in AgNPs FTIR spectrum represented the existence of C–N stretching vibration bond of aromatic amines^47^. The existence of peak at 1085 cm^−1^ in the FTIR spectrum indicated C–O–H stretching bond in primary and secondary alcohols^48^. The bands at 986 cm^−1^ and 864 cm^−1^ referring to the C–N and N–H stretching vibration bonds; respectively of primary amines suggesting the primary amine groups may involved in NPs synthesis^49^ and could present in capping agents of synthesized NPs. The peaks at rang of 561–568 cm^−1^ represent the vibration peaks of PO_4_^3−^ as reported by Maisara *et al*.^50^. Besides, the existence of weak band at 648 cm^−1^ is referring to –C≡C–H of stretch vibrations in alkynes. The bands at 520 and 405 cm^−1^ is referring to the existence of naphthalene and S–C≡N bend in thiocyanates.

Gole *et al*.^51^ emphasizes that proteins can bind nanoparticles by attracting a negative charge of carboxylate groups in the enzymes found in the culture supernatant or through cysteine residues or free amine groups in the proteins. This suggestion is coordinate with our results which confirmed the highly stability of the biosynthesized AgNPs provided by the surrounded proteins. Sastry *et al*.^52^ also illustrated that the functional groups such as –C–O–C–, –C = C–and –C–O– are found in heterocyclic compounds as proteins, that are present the capping ligands of the nanoparticles^53^. The presence of protein on the surface of the biosynthesized AgNPs was supported by FTIR analysis, confirming that proteins acting as a capping agent and prevents ingathering of the reduced AgNPs. “In fact, the carbonyl groups from amino acid residues as well as peptides of proteins are known for strong silver binding property. It has been suggested that stability of the AgNPs generated using cell-free culture supernatants could be due to the presence of a proteinaceous capping agent that prevents aggregation of the nanoparticles^54^”.

#### Zeta potential distribution of the biosynthesized AgNPs

**Figure 8:**
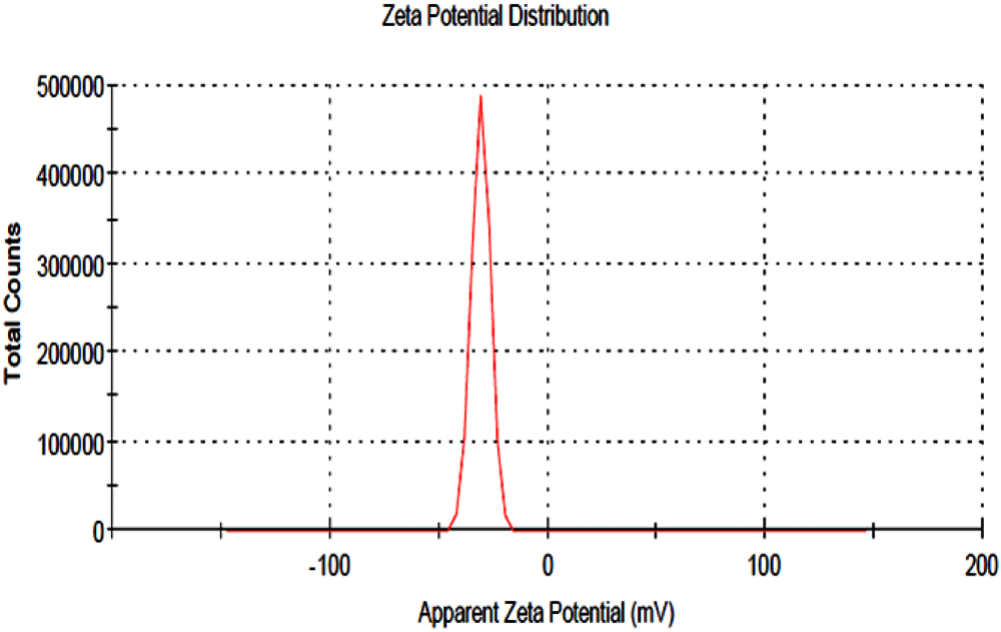
The zeta potential distribution graph showing negative zeta potential value for silver nanoparticles synthesized by using phycoerythrin pigment.

Zeta potential is a measure of the particle’s stability. The biosynthesized AgNPs have a Zeta potential measurement (Fig. 8) equals − 32.0 mV with SD “standard deviation” equals 4.93 mV and conductivity equals 0.722 mS/cm. Nanoparticles with zeta potential signal greater than + 30 mV or less than ‒30 mV are considered strongly cationic and strongly anionic; respectively^55^. The negatively charged Zeta potential measurements (−35.05 mV) of polysaccharides participated in the electrostatic stability of biosynthesized AgNPs^56^. Intensive signal at −32.2 mV revealed that the biosynthesized nanoparticles have negative charges on their surfaces^57^.

#### X-ray diffraction (XRD)

**Figure 9:**
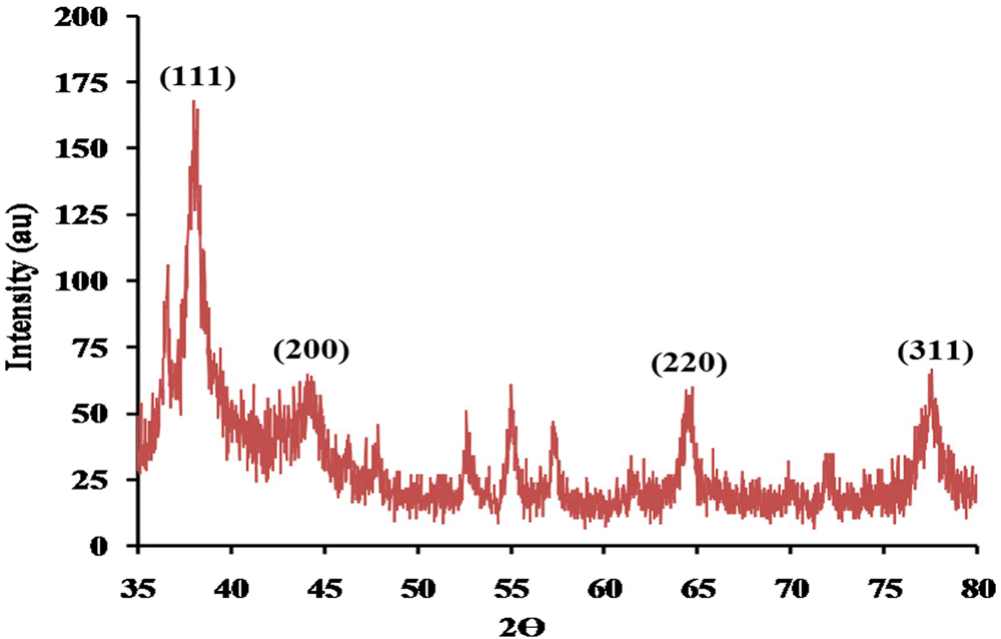
X-ray diffraction for silver nanoparticles synthesized by using phycoerythrin pigment.

XRD signals for the biosynthesized AgNPs are shown in Fig. 9. X- ray diffraction at 2θ in the range of 35 to 80° obviously detected well four diffraction signals (planes) appearing as 37.2°, 43.8°, 64.1°, and 77.6° at 2 theta (degree), that matched to the (111), (200), (220), and (311) planes of face centered cubic (fcc) structure silver; respectively. The analyzed four signals of the biosynthesized AgNPs prove that AgNPs is crystalline in nature. The size of the AgNPs crystals was calculated from the highest intensive signal (111) and was found to be 6.85 nm. XRD provides a perfectible illustration to the structure of the AgNPs because it shows axes, shape, size and position of the particles.

#### Stability and dispersion of the synthesized AgNPs

**Figure 10:**
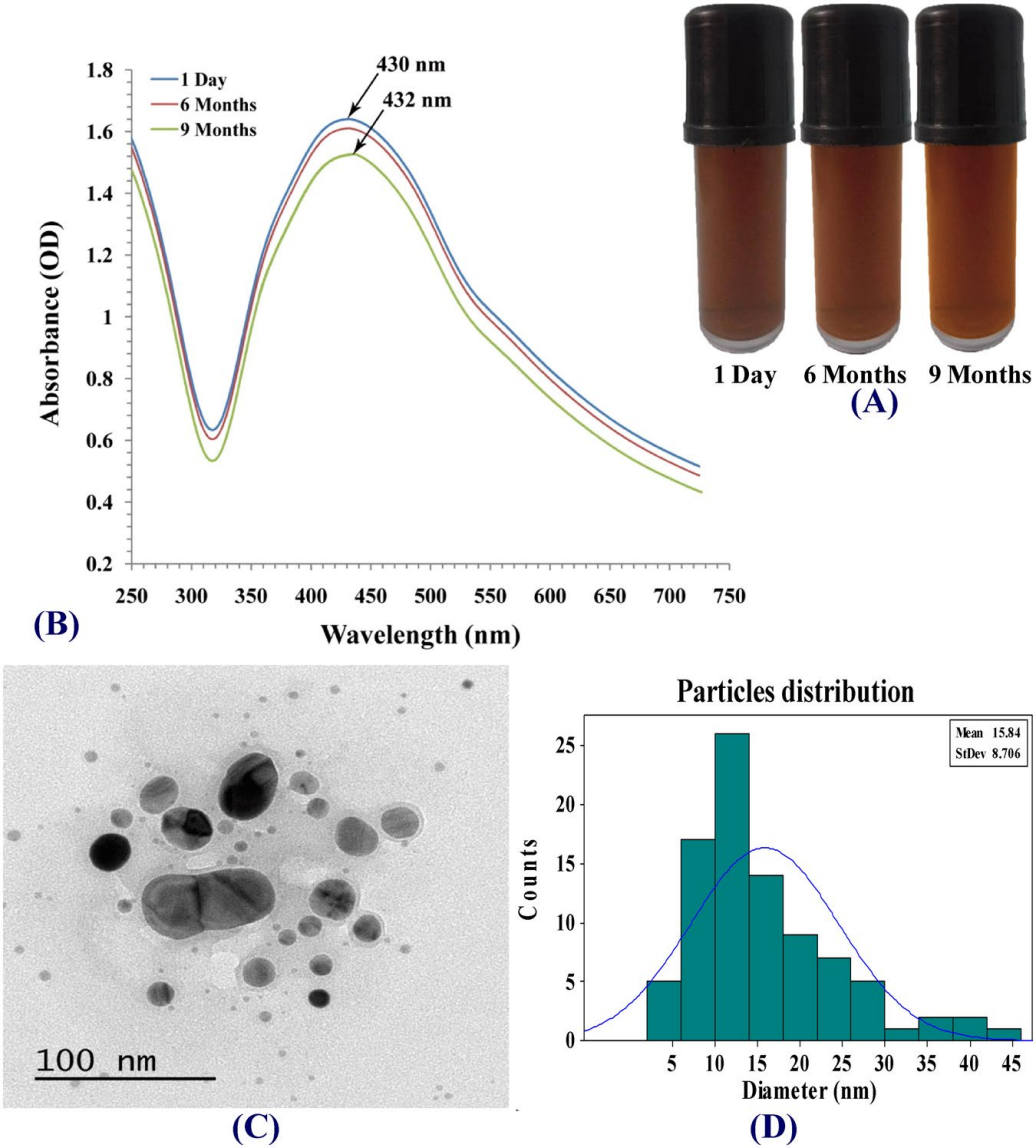
Stability and dispersion of synthesized silver nanoparticles. (**A**) Visible observation of AgNPs biosynthesis; (**B**) UV–Vis absorption spectrum of AgNPs; (**C**) TEM image of stored AgNPs; (**D**) Particle size distribution after a period of 6 months of synthesis.

The stability and the degree of dispersion of the synthesized AgNPs was studied by keeping AgNPs under dark condition in closed bottle for 1 day, 6 months and 9 months. It has been observed that, there is no significant change in color or appearance of agglomeration over a period of 6 months (Fig. 10A). The plasmonic properties characterized by UV-visible spectroscopy. UV-vis spectra indicated a shift in the peak position of the surface plasmon resonance from 430 to 432 nm with the stored AgNPs on 9^th^ month (Fig. 10B). Also, size and morphology of synthesized AgNPs were validated using transmission electron microscopy (TEM). TEM images presented in Fig. 5 for synthesized AgNPs after 1 day of synthesis and in Fig. 10C for stored AgNPs revealed that the AgNPs were well dispersed and there was no significant alteration in the size and the shape of AgNPs and no agglomeration tendency was noticed for a period of 6 months. So we can consider that the AgNPs show better long-term stability. The dispersion stability of the AgNPs after a period of 6 months was further analyzed by quantitative analysis of particle size distribution using Minitab 16 Statistical Software to calculate the mean size of particles which was found to be 15.84 (Fig. 10D).

The presence of a proteinacous compounds surrounded the surface of the biosynthesized AgNPs was confirmed by FTIR. AgNPs stability might be because of the existence of these proteinaceous compounds, acting as capping agents, which can bind to silver nanoparticles and prevents its aggregation^30^.

#### Antibacterial efficiency of AgNPs

**Figure 11:**
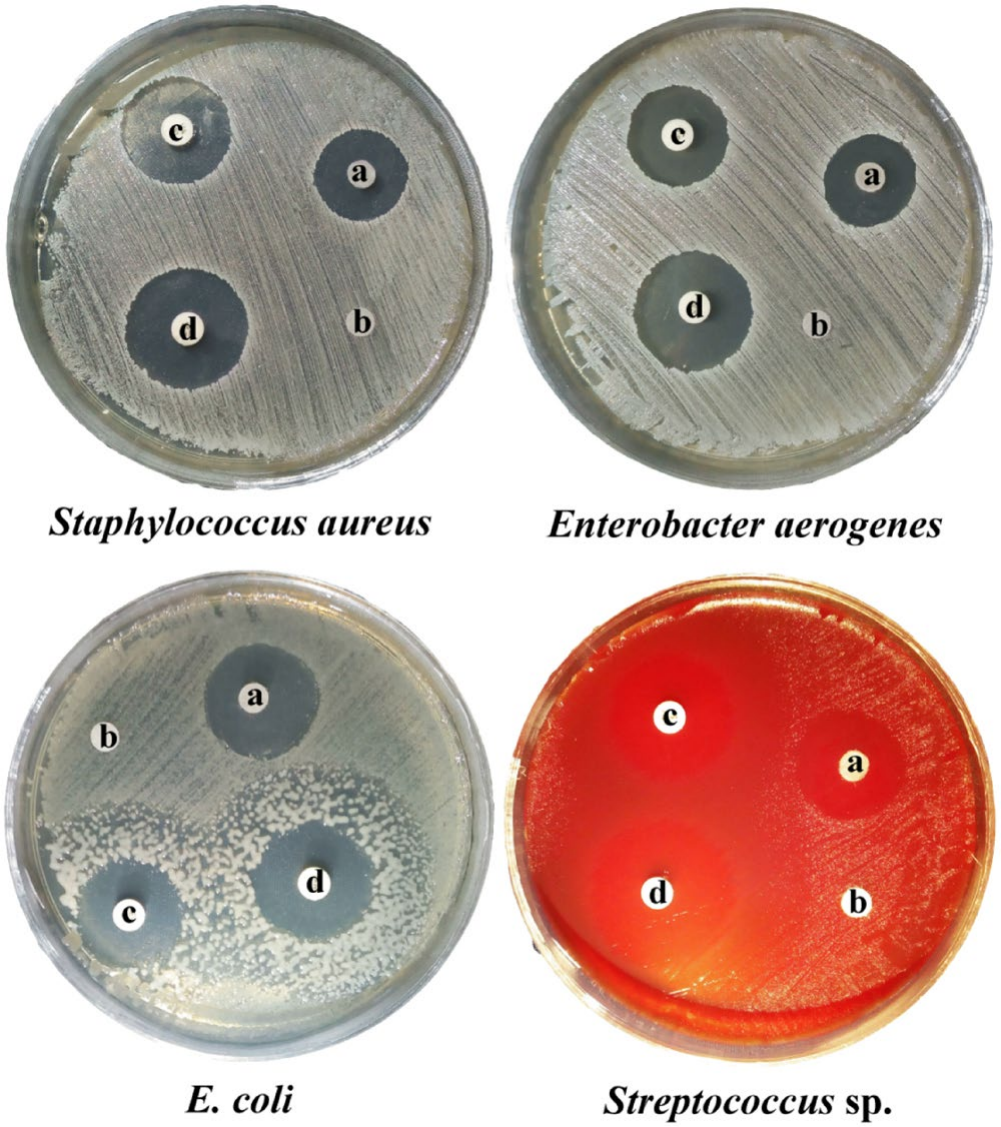
Antibacterial activity of silver nanoparticles produced by using phycoerythrin pigment against bacterial species where (**a**) AgNPs, (**b**) AgNO_3_ (20 mM) as control, (**c**) Vancomycin as positive control and (**d**) AgNPs + vancomycin.

In this study, the antibacterial efficiency of AgNPs was assessed against *Staphylococcus aureus* and *Streptococcus* sp. (G + ve bacteria), in addition, *Enterobacter aerogenes* and *E. coli* (G − ve bacteria) using the method of disc-diffusion as shown in Table 4 and Fig. 11. AgNO_3_ solution with 20 mM concentration was used as control. The highest antibacterial efficiency was noticed against *E. coli* (21 mm of inhibition zone) while a lower efficiency was noticed against *Staphylococcus aureus* (16 mm of inhibition zone). The diameter of inhibition zones against *Streptococcus* sp. and *Enterobacter aerogenes* were 18 mm which are larger than the inhibition zones obtained by Metuku *et al*.^58^ who tested the antibacterial efficiency of AgNPs against *Staphylococcus aureus* (15 mm inhibition zone) and *Enterobacter aerogenes* (16 mm inhibition zone). Addition of AgNPs to vancomycin improves the inhibitory influence towards the tested bacteria (Table 4). The antibacterial efficiency increased with increasing Ag^+^ concentration in AgNPs dispersion^59^. The proposed mechanism by which AgNPs exhibited antibacterial efficacy suggested that the smaller size AgNPs attach and penetrate the cell walls of bacteria causing cell membrane deformation and also interact with compounds which contains phosphorus and sulfur like DNA and causes disruption of DNA replication and finally destroying the bacterial cells^60,61^. Braydich-Stolle *et al*.^62^ documented that the AgNPs cause plasma membrane laceration and depletion of intracellular ATP leading to respiration block and cell destruction. The destruction of the bacterial cells may be due to modifying of the cellular signaling caused by AgNPs^63^.

#### Anti-hemolytic efficiency of AgNPs

The destruction in erythrocytes and the release of their contents (cytoplasm or hemoglobin) into blood plasma defined as hemolysis. Hemolysis inside the body can be caused by the action of bacteria, some autoimmune disorders like drug-induced hemolytic anemia, some genetic disorders like sickle-cell disease, or blood with low solute concentration (hypotonic to the cells). The degree of erythrocyte damage is so high because of the disintegration of the structural integrity of the lipid layer at a specific concentration of 2, 2ʹ-azobis (2-amidinopropane) dihydrochloride (AAPH) which is consider a peroxyl radical initiator that release free radicals by its thermal decomposition and will attack red blood corpuscles for induction of multiple side-chain oxidations of protein and lipid causing successive changes in their membrane structure that lead to hemolysis^64^.

In the present study, AAPH solution was used to induce complete erythrocyte hemolysis (100%) of blood obtained from rats. The hemolysis percentage was decreased when the reaction mixture was treated with AgNPs. Silver nanoparticles show an anti-hemolytic activity with inhibition of 96.4% (erythrocyte hemolysis was reduced to be 3.6%) which was comparable to that of vitamin C that exhibit anti-hemolytic activity with 96% inhibition. The proposed mechanism for anti-hemolytic potential of silver nanoparticles is due to those the capping agents that surrounded silver nanoparticles involve amino compounds (-NH_2_) which have anti-hemolytic properties^65^ and also Ar-OH phenols “the functional group of phenolic compounds” that have anti-oxidant properties against radicals depending on the similarity of their redox characters^66^.

#### *In vitro* anti-cancer activity

**Figure 12:**
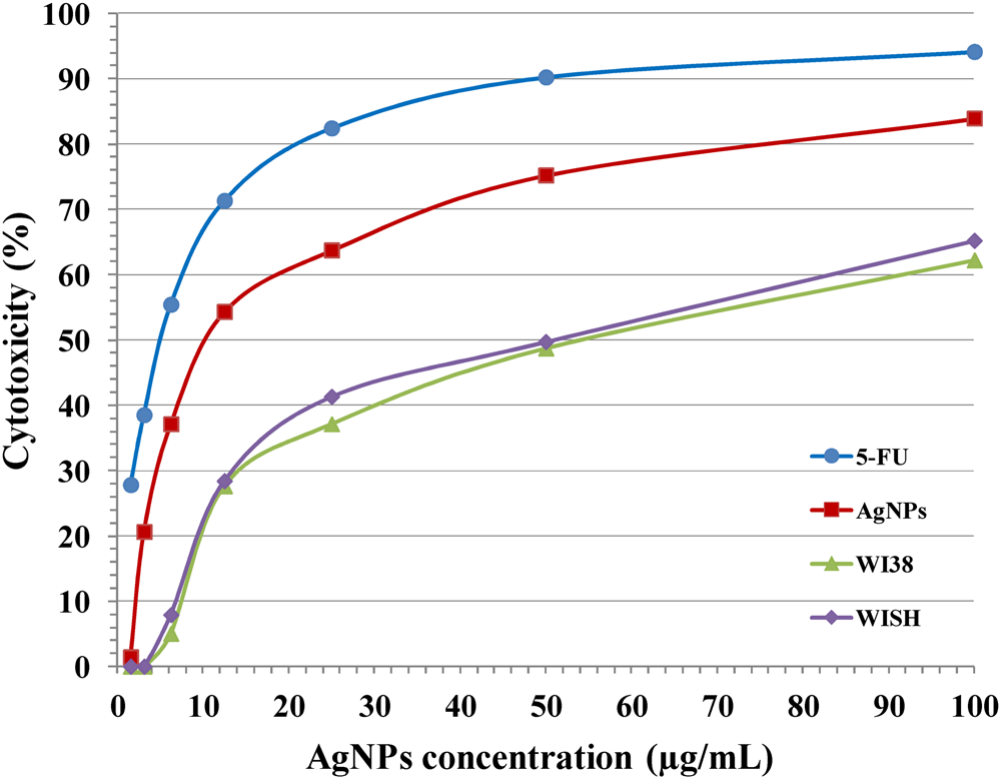
*In vitro* anti-cancer activities of various concentrations of AgNPs on mammary gland breast cancer cell line (MCF-7), human lung fibroblast (WI38) and human amnion (WISH) cell lines. 5-fluorouracil was used as a standard anticancer drug for comparison.

The cell line death rate of MCF-7 is demonstrated in Fig. 12 which increases with increase in AgNPs concentration. The IC_50_ (the half maximal inhibitory concentration) value is the concentration of AgNPs required to produce cell growth inhibition of MCF-7 cell line by 50% after 48 hours of incubation compared to untreated controls. The IC_50_ of the biosynthesized AgNPs was recorded at 13.07 ± 1.1 µg/mL, which is strong compared to IC_50_ of 5-FU (standard) observed at 4.81 ± 0.3 µg/mL (very strong). Also after 48 h of incubation of the biosynthesized AgNPs with WI38 and WISH “normal cell lines”, the IC_50_ of them were recorded at 45.76 ± 2.6 (moderate) and 52.13 ± 3.1 (weak) µg/mL; respectively which is compared to the IC_50_ of 5-FU (standard) against these normal cell lines, which were recorded at 6.68 ± 0.5 and 5.52 ± 0.4 µg/mL (very strong); respectively. Therefore, it was detected that the cytotoxic effect of the biosynthesized silver nanoparticles against MCF-7 was stronger than on WI38 and WISH normal cell lines. While 5-FU cytotoxicity against WISH and WI38 normal cell lines is stronger than on breast cancer cells. This means that the use of AgNPs to treat breast cancer is better than using the 5-FU as its toxic effect on normal cells is much lower. AgNPs have cytomorphological changes on MCF-7 cells as oxidative stress, cell shrinkage, involution and biochemical reactions resulted in apoptosis^67^. AgNPs can increase oxidative degradation of lipids and resulted in DNA deterioration, necrosis and apoptosis^68,69^. AgNPs is considering effective photothermal agent in cancer therapy that induced hyperthermia within the breast cancer cells^70^. The apoptosis may be occurred through the dysfunction of mitochondria resulting in the proliferation inhibition for MCF-7 cells^71^.

Despite the promising advantages of AgNPs, safety concerns have been raised on the use of AgNPs because they pose potential toxicity in living organisms. Many *in vitro* studies showed the ability of AgNPs to induce toxicity administrated by increased production of reactive oxygen species, apoptosis, DNA damage, proinflammation, etc.^72^.

#### *In vivo* anti-cancer efficiency

Table 5 represented the anti-cancer efficiency of silver nanoparticles in EAC (Ehrlich ascites carcinoma) bearing mice including the complete blood count parameters, tumor volume, cells count and body weight. Healthy mice have hemoglobin (HB) content equalled 13.53 ± 1 g/dl and RBCs count equaled 5.23 × 10^6^/mm^3^. However, EAC bearing mice showed lower values; HB was 8.10 ± 1 g/dl and RBC was 3.36 × 10^6^/mm^3^, indicating the existence of anemia which is the most considerable dilemma in treating cancer, which probably happened because of decreasing in iron level, blood haemolysis or acute spinal cord injury^73^. In addition, the healthy mice have WBCs count equalled 4.97 ± 1 × 10^3^/mm^3^ and body weight equaled 24.2 g. However, EAC bearing mice showed higher values; WBCs count became 15.06 ± 1 × 10^3^/mm^3^ and body weight became 37.7 ± 3 g. This elevation in WBCs count is due to the intensive defense against the cancer as well as the elevation in the body weight is due to the increase in tumor volume, which equaled 7.6 ± 1 mL and the tumor cells count, which equaled 49.15 ± 10 × 10^6^/mL.

**Table 5:** Effect of AgNPs and 5-FU on hematological parameters, tumor parameters (volume and cell count) and body weight of EAC bearing mice.

Group	Hb (g/dl)	RBC count 10^6^/mm^3^	Total WBC 10^3^/mm^3^	Tumor cells count (10^6^/mL)	Body Wt. (g)	Tumor volume (mL)	ΔT/ΔC (%)	% Inhibition
Normal control	13.53 ± 1	5.23	4.97 ± 1	—	24.2 ± 2	—	—	—
EAC control	8.10 ± 1	3.36	15.06 ± 1	49.15 ± 10	37.7 ± 3	7.6 ± 2	100	0
5-FU	12.97 ± 1	4.52 ± 1	9.32 ± 1	13.24 ± 7	28.6 ± 2	1.5 ± 1	19.74	80.26
AgNPs	13.12 ± 1	4.98	7.11 ± 1	11.76 ± 7	26.1 ± 2	1.2 ± 1	15.79	84.21

After the administration of 5 mg AgNPs/kg of mice body weight, HB content elevated to be 13.12 ± 1 g/dl, RBCs count also elevated to be 4.98 × 10^6^/mm^3^, WBCs count decreased to 7.11 ± 1 × 10^3^/mm^3^, tumor cells count decreased to 11.76 ± 7 × 10^6^/mL, body weight decreased to 26.1 ± 2 g and tumor volume decreased to 1.2 ± 1 mL. Compared with EAC control mice, tumors growth was significantly inhibited in the EAC bearing mice injected with AgNPs-treated group (Table 5) by 84.21% which is better than the result obtained with 5-FU-treated group (tumor growth inhibition of 80.26%) as showing in Table 5. The obtained results were compatible with the results obtained by Antony *et al*.^74^ which indicate the efficiency of AgNPs in inhibition of EAC growth in the experimental animals. The efficiency of AgNPs in inhibition of EAC growth in the treated mice can be explained by the smaller size of the biosynthesized AgNPs ranged between 7.1‒26.68 nm which have the ability to disperse and accumulate in tumor cells after injections in the mice. The anti-cancer efficiency of AgNPs is contrariwise proportionate to its size^75^. Tumor cells lost their ability to divide in a controlled fashion, so tumors induce blood vessel growth from pre-existing vessels (angiogenesis) to compensate their needs from nutrients and oxygen^76^. The ascetic fluid supplied the tumor cells with nourishment needed for growing, so it was suggested that the treatment with AgNPs suppress the angiogenesis process and consequently decreased the EAC nourishment and arrest tumor growth through decrease in nutritional fluid volume^77^. El Bialy *et al*.^78^ have previously reported that AgNPs possessed anti-cancer efficiency against Ehrlich Cell Carcinoma in mice due to its potential oxidative damage effect proved by elevation of malondialdehyde (MDA) and H_2_O_2_ contents in the solid tumor tissue as indicative for lipid peroxidation and free radicals production in tumor tissue and induction of apoptosis via caspase 3 activation.

Despite the obvious benefits of AgNPs, their use in therapeutic or diagnostic application generates concerns about their safety for human health because they induce serious risks in humans and in other living organisms. However, *in vivo* research has attempted to characterize the toxicity features such as pulmonary toxicity, neurotoxicity, reproductive toxicity, etc. and to determine the lethal dose, 50% (LD_50_) of AgNPs^72^. The ways in which AgNPs enter the human body are injection in blood circulation, skin penetration through lesions or scratch, oral ingestion or pulmonary inhalation. In addition to exposure pathway, dose and duration are identified as important determinants affecting biodistribution and pathogenic outcomes that cause toxic exposure^79^. Guo *et al*.^80^ assessed the *in vivo* toxicity of AgNPs administered with single or multiple intravenous injections in female Balb/c mice for one, four and 10 days. They showed the distribution and toxicity of AgNPs caused by the destruction of the endothelial barrier in the liver, lungs and kidneys. The *in vivo* effect of AgNPs has been explored in the food of mice, rats and weaned pigs. Fondevila *et al*.^81^ reported that AgNPs contributed to a distinct increase in the body weight of pigs depending on the dose after oral ingestion for 14 days but did not have an effect on the mucosa of the pig ileum. In addition, Mao *et al*.^72^ reported that oral ingestion of AgNPs in animals has shown that AgNPs are able to distribute to most organs and dietary AgNPs caused a variety of ROS mediated stress responses, including apoptosis, DNA damage, and autophagy in *Drosophila melanogaster*. Many research efforts aim at surface modification of NPs to make them safer and less toxic, to determine the dose at which nanomaterials can be considered safe and therefore suitable for daily consumer products^82^.

## Materials and Methods

### Cyanobacterial isolate and cultural conditions

*Nostoc carenum* was isolated from soil sample collected from garden at El-Dakahlia Governorate, Egypt. *Nostoc carenum* was grown for 21 days in axenic culture at 28 ± 2 °C under continuous illumination of 3200 lux in conical flasks (500 mL) containing 250 mL of BG-11 medium^83^, pH was adjusted at 7.

### Extraction and purification of phycoerythrin

Phycoerythrin was extracted from *Nostoc carneum* using the method described by El-Naggar *et al*.^84^ then the debris of the cells were removed by centrifugation for 10 min. at 5000 × *g*. Purification of the extract was done through a single step of precipitation by 65% of ammonium sulphate (NH_4_)_2_SO_4_ and kept overnight at 4 °C. The precipitate was separated by centrifugation at 4 °C for 15 min at 27000 × *g*, then the precipitate resuspended in 10 mL of the phosphate buffer^85^.

### Biosynthesis of AgNPs

AgNPs were biosynthesized by adding 19 mL (20 mM) of AgNO_3_ solution to 1 mL of phycoerythrin. The pH of the reaction mixture has been adjusted to pH 10 and kept inside a “closed system under direct lighting 2400–2600 Lux at room temperature” according to the modified method of Patel *et al*.^11^. Changing the color from pink to dark brown color indicate the formation of silver nanoparticles which measured by UV– vis spectroscopy in the range of 200–800 nm using AgNO_3_ solution as control. AgNPs were centrifuged using “MIKRO 120 Hettich Zentrifugen D-78532 Tuttlingen Germany at 10,000 × *g* for 10 min”. The supernatant was thrown away; the silver nanoparticles precipitate was obtained, washed ten times with distilled H_2_O and freeze-dried.

To quantify the experimental silver nanoparticles concentration in face-centered central composite design, the absorbances of synthesized AgNPs solutions were measured using ultraviolet-visible spectroscopy (UV-vis) at 430 nm. Standard curve was made by serial dilution of 2 mg/mL of lyophilized AgNPs synthesized under the optimum conditions. Values of silver content in the prepared solutions were obtained by using the equation for a straight line:

2 $${\rm{Y}}={\rm{mx}}+{\rm{b}}$$

where m is the slope and b the intercept.

### Face-centered central composite design (FCCD)

FCCD was used to optimize and study the effect of AgNO_3_ concentration, phycoerythrin pigment concentration, incubation period and initial pH level on biosynthesis of the AgNPs. “FCCD is an effective design that is used for sequential experimentation and provides reasonable amount of information for testing the goodness of fit and does not require large number of design points thereby reducing the overall cost associated with the experiment^86^”. The four tested independent factors were studied using 30 runs. Each factor was used at three levels (−1, 0, 1). “The central point was repeated six times” to provide the estimation of the pure error and predict lack of fit of the model. All runs were performed in duplicate and the average obtained for silver nanoparticles was taken as the dependent variable (Y). The experimental results of FCCD were fitted using the following “second order polynomial equation:

3 $$Y={\beta }_{0}+\sum _{i}{\beta }_{i}{X}_{i}+\sum _{ii}{\beta }_{ii}{X}_{i}^{2}+\sum _{ij}{\beta }_{ij}{X}_{i}{X}_{j}$$

In which Y is the predicted synthesized AgNPs, β_0_, β_i_, β_ii_, β_ij_ are the regression, linear, quadratic and interaction coefficients; respectively and X_i_ is the independent factors coded levels.

### Statistical analysis

The experimental designs and statistical analysis were performed using the software Design Expert version 7 for Windows and to plot the 3-D surface plots, the STATISTICA software “Version 8.0, StatSoft Inc., Tulsa, USA” was used.

### Characterization of AgNPs

#### UV–vis absorbance of AgNPs

UV-vis spectrophotometer “ATI Unicam 5625 UV/VIS Vision Software V3.20 at Spectrum Unit of Faculty of Science, Mansoura University, Egypt” used to monitor silver nanoparticles concentrations and solution of silver nitrate was used as a control, the absorption spectrum was scanned in the range of wavelength from 200 to 800 nm. “UV–visible spectra have proven to be very effective for analyzing of different nanoparticles such as gold and silver nanoparticles^87^”.

#### TEM examinination of AgNPs

A drop of the washed silver nanoparticles was dispersed onto copper grid, dried, coated with carbon and examined using “JEOL-JEM-100 CXII operating at an accelerating voltage of 200 kV at Electron-Microscope-Unit of Mansoura University, Egypt” to determine the size and shape of silver nanoparticles^88^.

#### EDX analysis

EDX analysis is an analytical technique used for the elemental analysis or chemical characterization of the synthesized silver nanoparticles by a field-emission of scanning electron microscope^89^ using “Oxford X-Max 20 Instrument at Electron Microscope Unit, Mansoura University, Mansoura, Egypt.”

#### FTIR analysis

The functional groups responsible for reduction of silver ions and stabilization of silver nanoparticles were detected using “FTIR instrument (Thermo Scientific Nicolet iS10 FT-IR spectrometer) At Spectrum Unit of Faculty of Science, Mansoura University, Mansoura, Egypt”. The analysis was performed using AgNPs-KBr pellets (in the ratio of 1:100). “The spectrum was recorded over a spectral range from 500 to 4000 cm^−1^ and the spectral resolution was 4 cm^−1^”according to the method of Singh *et al*.^90^.

#### Zeta potential analysis for AgNPs

Surface charge and size distribution of silver nanoparticles were detected using “particle size analyzer (Zeta sizer nano ZS90, Malvern Instruments Ltd., U.K.) at 25 °C with 90° detection angle at Electron Microscope Unit of Mansoura University, Mansoura, Egypt”. Zeta potential is consider the net surface charge of AgNPs^91^.

#### XRD pattern of AgNPs

XRD can give information about crystal structure, phase and texture of AgNPs through X-ray beam “with monitoring the diffraction angle from 5° to 80° (2ϴ)^92^ Philips X’pert Pro, Panalytical”. The average size of AgNPs was calculated by the use of full width at half maximum (FWHM) of face-centered cubic (111) using the Debye–Scherrer equation,

4 $$K\lambda /\beta \,cos\,\theta $$

“where *K* is the Scherrer constant with value from 0.9, *λ* is the wavelength of the X-ray, *β* is the full width at half maximum and *θ* is the Bragg angle in radians”.

#### Stability and dispersion of AgNPs

For guarantee the dispersion stability of AgNPs in aqueous dispersion, it was kept under dark condition in closed bottle for 1 day, 6 months and 9 months. The plasmonic properties characterized by UV-visible spectroscopy. Size and morphology were validated using Transmission Electron Microscopy. The particle size distribution was validated by quantitative analysis using Minitab 16 Statistical Software.

#### Antibacterial efficiency of AgNPs

Antibacterial efficiency of AgNPs was assessed against *Staphylococcus aureus* and *Streptococcus* sp. (G + ve bacteria), in addition, *Enterobacter aerogenes* and *E. coli* (G − ve bacteria) using the method of agar disc-diffusion^93^. Sterile filter paper discs were loaded with 60 µl of the dispersion of 1000 µg/mL silver nanoparticles; the control disc was loaded with the solution of 20 mM AgNO_3_. 30 µg vancomycin disks also have been loaded with the same concentration of silver nanoparticles. The inoculated plates were incubated for 24 h at 37 °C and then examined for inhibition zones existence. The inhibition zones diameters surrounding the discs were measured in millimeters.

#### Anti-hemolytic efficiency of silver nanoparticles

Silver nanoparticles anti-hemolytic efficiency was detected according to the method of El-Naggar *et al*.^84^. Hemolysis of rats’ erythrocytes was initiated by peroxyl free radicals toxicity induced by “2,2′-azobis (2-amidinopropane) dihydrochloride (AAPH) solution^94^. Vitamin C (L- ascorbic acid) was used as a positive control”.

#### *In vitro* anti-cancer efficiency of silver nanoparticles

*In-vitro* cytotoxic efficiency of silver nanoparticles was assessed against “breast cancer (MCF-7), human lung fibroblast (WI38) and human amnion (WISH)” cell lines that were attained from VACSERA “ATCC via holding company for biological products and vaccines, Cairo, Egypt” using MTT assay “3-(4, 5-Dimethyl thiazol-2yl)-2, 5-diphenyl tetrazolium bromide” colorimetric technique^95^ and the modified method of El-Naggar *et al*.^84^. AgNPs or 5-fluorouracil as standard with same concentrations (1.56, 3.125, 6.52, 12.5, 25, 50, and 100) was inoculated into the medium containing the grown cell lines. Percentage of the cytotoxicity was calculated by using the next equation:

5 $$\begin{array}{c}{\rm{Viability}} \% =({\rm{OD}}\,{\rm{of}}\,{\rm{the}}\,{\rm{Test}}/{\rm{OD}}\,{\rm{of}}\,{\rm{the}}\,{\rm{control}})\times 100\\ {\rm{Cytotoxicity}} \% =100-{\rm{Viability}} \% \end{array}$$

#### *In vivo* anti-cancer efficiency

Ethics statement: “All experimental protocols were approved by Research Ethics Committee, Faculty of science, Mansoura University, Mansoura, Egypt. All the experiments were performed in accordance with the relevant guidelines and regulations”.

Adult Swiss albino male mice (weight, 20–25 g) were attained from “Pharmacology Department, Mansoura University, Egypt and the Ehrlich ascites carcinoma (EAC) cells were obtained from National Cancer Institute (NCI), Cairo, Egypt”. They are housed in a controlled environment “temperature 25 ± 2 °C” and 12 hours dark/light cycle with standard laboratory diet and water ‘*ad libitum*’. The Ehrlich ascites carcinoma (EAC) cells were “provided by National Cancer Institute (NCI), Cairo, Egypt. EAC cells were diluted with normal saline (0.9% NaCl) to reach the desired concentration (2 × 10^6^ cells/0.2 mL).The mice were divided into four groups comprising five animals in each group. Group I represents negative control-received vehicle (normal saline) with no EAC cells; group II served as tumor control injected with EAC (2 × 10^6^ cells per mouse); group III represents EAC bearing mice inoculated with 5-FU; group IV represents EAC bearing mice inoculated with AgNPs. So all groups were injected intraperitoneally with 2 × 10^6^ cells per mouse except group I. After 24 h, mice were injected with the anti cancer 5-FU and AgNPs (5 mg/kg body weight). The inoculation process was repeated for 10 days. After 24 h of the last dose including 18 h of fasting half of the mice in each group were weighed and sacrificed. Blood samples were collected from each group of sacrificed mice for the estimation of hemoglobin (Hb) content and counts for red (RBC) and white blood cells (WBC) were performed. Tumor volume and tumor cells count were determined by collecting the ascetic fluid from the peritoneal cavity of the mice. The count of viable cells and non-viable cells was estimated by taking a part of the ascetic fluid then centrifugated and stained with trypan blue (0.4% in normal saline) and the other part was centrifuged in a graduate centrifuge tube at 1,000 rpm for 5 min and the packed volume was measured. The increase of mice body weights were recorded both in the treated and control groups at the start of the experiment to the end (0 to 11 days)”.

Anti-tumor effectiveness was evaluated by the estimation of the average change of tumor volume in the treatment group (ΔT) and the average change of tumor volume in the control EAC bearing mice (ΔC). “The degree of tumor growth was calculated as ΔT/ΔC × 100 which was then subtracted from 100% to estimate the percentage (%) of tumor growth inhibition”^96^.

## Conclusion

For the first time, silver nanoparticles were successfully synthesized by using phycoerythrin extracted from *Nostoc carneum* via eco-friendly green approach. Response surface methodology is an effective experimental method simplifies the optimization of multiple variables for expecting the perfect yield with a lowest number of trials and describes the individual and interaction effects of test variables on AgNPs synthesis to attain a higher AgNPs biosynthesis. After statistical optimization by response surface methodology, the AgNPs biosynthesis was improved (1789.341 µg/mL) as compared with that of unoptimized conditions (468.308 µg/mL) with fold of increase 3.82. AgNPs were characterized by FTIR, UV-vis, TEM, Zeta potential and the crystalline nature was confirmed by XRD. Based on the obtained data, it is proved that the biosynthesized AgNPs possess anti-hemolytic, antibacterial and anticancer efficiency of great potential for cosmetic, pharmaceutical, medicinal and therapeutic applications. Despite the promising advantages of AgNPs, safety concerns have been raised on the use of AgNPs because their bioaccumulation and toxicity in living organisms. More studies needs to be done to evaluate the bioaccumulation and toxicity of silver nanoparticles.
